# New mechanism of nerve injury in Alzheimer’s disease: β‐amyloid‐induced neuronal pyroptosis

**DOI:** 10.1111/jcmm.15439

**Published:** 2020-06-10

**Authors:** Chenyang Han, Yi Yang, Qiaobing Guan, Xiaoling Zhang, Heping Shen, Yongjia Sheng, Jin Wang, Xiaohong Zhou, Wenyan Li, Li Guo, Qingcai Jiao

**Affiliations:** ^1^ State Key Laboratory of Pharmaceutical Biotechnology School of Life Science Nanjing University Nanjing China; ^2^ Department of Pharmacy The Second Affiliated Hospital of Jiaxing University Jiaxing China; ^3^ Department of Neurology The Second Affiliated Hospital of Jiaxing University Jiaxing China; ^4^ Department of Center Laboratory The Second Affiliated Hospital of Jiaxing University Jiaxing China

**Keywords:** Alzheimer's disease, GSDMD, pyroptosis, β‐amyloid

## Abstract

The present study was designed to investigate the role of β‐amyloid (Aβ_1‐42_) in inducing neuronal pyroptosis and its mechanism. Mice cortical neurons (MCNs) were used in this study, LPS + Nigericin was used to induce pyroptosis in MCNs (positive control group), and Aβ_1‐42_ was used to interfere with MCNs. In addition, propidium iodide (PI) staining was used to examine cell permeability, lactate dehydrogenase (LDH) release assay was employed to detect cytotoxicity, immunofluorescence (IF) staining was used to investigate the expression level of the key protein GSDMD, Western blot was performed to detect the expression levels of key proteins, and enzyme‐linked immunosorbent assay (ELISA) was utilized to determine the expression levels of inflammatory factors in culture medium, including IL‐1β, IL‐18 and TNF‐α. Small interfering RNA (siRNA) was used to silence the mRNA expression of caspase‐1 and GSDMD, and Aβ_1‐42_ was used to induce pyroptosis, followed by investigation of the role of caspase‐1‐mediated GSDMD cleavage in pyroptosis. In addition, necrosulfonamide (NSA), an inhibitor of GSDMD oligomerization, was used for pre‐treatment, and Aβ_1‐42_ was subsequently used to observe the pyroptosis in MCNs. Finally, AAV9‐siRNA‐caspase‐1 was injected into the tail vein of APP/PS1 double transgenic mice (Alzheimer's disease mice) for caspase‐1 mRNA inhibition, followed by observation of behavioural changes in mice and measurement of the expression of inflammatory factors and pyroptosis‐related protein. As results, Aβ_1‐42_ could induce pyroptosis in MCNs, increase cell permeability and enhance LDH release, which were similar to the LPS + Nigericin‐induced pyroptosis. Meanwhile, the expression levels of cellular GSDMD and p30‐GSDMD were up‐regulated, the levels of NLRP3 inflammasome and GSDMD‐cleaved protein caspase‐1 were up‐regulated, and the levels of inflammatory factors in the medium were also up‐regulated. siRNA intervention in caspase‐1 or GSDMD inhibited Aβ_1‐42_‐induced pyroptosis, and NSA pre‐treatment also caused the similar inhibitory effects. The behavioural ability of Alzheimer's disease (AD) mice was relieved after the injection of AAV9‐siRNA‐caspase‐1, and the expression of pyroptosis‐related protein in the cortex and hippocampus was down‐regulated. In conclusion, Aβ_1‐42_ could induce pyroptosis by GSDMD protein, and NLRP3‐caspase‐1 signalling was an important signal to mediate GSDMD cleavage, which plays an important role in Aβ_1‐42_‐induced pyroptosis in neurons. Therefore, GSDMD is expected to be a novel therapeutic target for AD.

## 1 BACKGROUND

Alzheimer's disease (AD) is a neurodegenerative disease characterized by cognitive decline, which is the leading cause of dementia.^1^, ^2^ The pathological features of AD mainly include the senile plaque (SP) formed by the deposition of β‐amyloid (Aβ) in the extracellular space,^3^ neurofibrillary tangles (NFT) formed by the abnormally phosphorylated Tau protein aggregation, vascular amyloidosis and loss of neurons in the cortex and hippocampus.^4^ Aβ was first discovered by the German pathologist Virchow in 1853, which was confirmed to contain 39‐43 amino acids; the commonly used Aβ_1‐42_ contains all amino acid sequences, which relatively accurately simulates the action of Aβ.^5^ Relevant AD studies have shown that Aβ can induce neuronal apoptosis, induce oxidative stress, activate microglia to induce inflammatory response in the central nervous system (CNS), induce imbalanced neuronal calcium ion homeostasis and reduce membrane fluidity. Therefore, Aβ‐induced neuronal damage is multi‐dimensional and multi‐mechanism.^6^, ^7^

The inflammatory response is a defence mechanism of the human body and an important physiological and pathological process against injury and infection; additionally, inflammatory response has been found to play an important role in various neurological diseases.^8^, ^9^ Studies on AD have revealed the aggregation of glial cells around the SP in the brain tissue of patients and AD rats, subsequently causing inflammation, and the abnormally elevated expression of inflammatory factors, including interleukin‐1 (IL‐1), IL‐6 and tumour necrosis factor‐α (TNF‐α), which further amplifies the inflammatory cascade.^10^, ^11^ Pyroptosis is a recently identified new type of cell death mediated by caspase family, which is characterized by the opening of cell membrane pores, swelling of cell membranes and release of inclusions, during which inflammatory factors are abundantly expressed and further released into the surrounding environment through open cell membrane pores to increase the local inflammatory factor levels.^12^, ^13^, ^14^

Although Aβ has been found to induce neuroinflammatory responses, central inflammation is mainly mediated by microglia.^8^ However, it remains uncovered whether Aβ directly causes inflammatory injury of nerve cells as well as the underlying mechanisms. In view of the role of pyroptosis in inflammatory response, in the present study, we investigated whether Aβ directly caused inflammatory injury of nerve cells as well as the underlying mechanisms, to further reveal the role of Aβ in AD‐related inflammatory responses.

## 2 MATERIALS AND METHODS

### 2.1 Mouse cortical neurons (MCNs) culture and induction of pyroptosis

Mouse cortical neurons (Procell) were cultured in mouse cortical neuron complete medium (Procell). MCNs were grouped when cells reached to about 70% confluency. MCNs were routinely cultured in the control, LPS + Nigericin and Aβ_1‐42_ groups, while MCNs were induced to undergo pyroptosis in LPS + Nigericin group. Cells were pre‐treated with 1 μg/mL of LPS (Sigma) for 4 hours, followed by incubation with 10 μmol/L of Nigericin (MCE)) to induce pyroptosis. In addition, MCNs were treated with 25 μmol/L Aβ_1‐42_ (Sigma).

To determine whether Aβ_1‐42_ induced pyroptosis by caspase‐1‐GSDMD, cells were treated with siRNA targeting caspase‐1 and GSDMD (GenePharma Co., Ltd.). Cells were divided into Aβ_1‐42_, siRNA‐caspase‐1 and siRNA‐GSDMD groups. MCNs in the Aβ_1‐42_ group were incubated with 25 μmol/L Aβ_1‐42_, and MCNs were transfected with siRNA‐caspase‐1 and siRNA‐GSDMD.

To determine whether Aβ_1‐42_ induced pyroptosis activating GSDMD oligomerization, the p30‐GSDMD oligomerization inhibitor, necrosulfonamide (NSA) (MCE, Shanghai, China) was used for intervention. Cells were divided into two groups, namely Aβ_1‐42_ and Aβ_1‐ 42_ + NSA groups. MCNs in the Aβ_1‐42_ group were treated with 25 μmol/L Aβ_1‐42_, while MCNs in the Aβ_1‐42_ + NSA group were pre‐treated with 20 μmol/L NSA for 2 hours, followed by incubation with 25 μmol/L Aβ_1‐42_.

### 2.2 Intervention and grouping of APP/PS1 mice

APP/PS1 double transgenic mice are commonly used AD model mice, with AD‐like behavioural alleviation at 3‐4 months of age and with AD‐like lesions at 1‐3 months of age. In our study, 4‐month‐old mice (purchased from the second hospital of Jiaxing University) were used, and 10 mice were divided into control and siRNA‐caspase‐1 groups. AAV9‐siRNA‐caspase‐1 is a central nervous‐specific transfected adeno‐associated virus, and siRNA‐caspase‐1 was mediated by AAV. In brief, mice were anesthetized with 10% chloral hydrate, and alcohol and iodophor were used to wipe the mouse tail. Afterwards, the tail of the mouse was placed in warm water, followed by injection of viral solution (200 μL) into the root of the tail. In addition, normal saline was injected with the equal volume in the control mice. After injection, mice were maintained in the cage.

### 2.3 Transfection of siRNA

siRNA (GenePharma Co., Ltd.) was used to silence GSDMD, and caspase‐1. MCNs were seeded into 24‐well plates at confluency of 30%. The total medium before transfection was 0.45 mL. A 50 pmol (0.67 μg) of siRNA was added to Opti‐MEM (Sigma) at a final volume of 25 μL. One microliter of Entranster‐R4000 was added to 24 μL of Opti‐MEM, followed by incubation at room temperature for 5 minutes. The diluted siRNA and the diluted Entranster‐R4000 dilution were mixed for incubation at room temperature for 15 minutes. Fifty microliter of the transfection solution was added to 0.45 mL of complete medium to mix well. After transfection for 6 hours, the state of MCNs was good, and MCNs were incubated for 48 hours.

### 2.4 Detection of cytotoxicity and membrane permeability changes

1. Lactate dehydrogenase cytotoxicity assay: LDH kit (Solarbio) was purchased for detecting cytotoxicity. MCNs were treated with Nigericin to induce pyroptosis and incubated with Aβ_1‐42_ for 2 hours, followed by detection of LDH release rate. Results were shown as (%).
2. Propidium iodide (PI) absorption rate assay: The cell permeability of pyroptotic cells was increased, and PI can penetrate into the cell membrane pore for staining; therefore, the cell permeability can be detected by the relative absorption rate of PI. Within 2 hours after Nigericin and Aβ_1‐42_ intervention, PI absorption rate was detected every 10 minutes (a total of 12 time points). In brief, 1 μg/mL of PI, 120 nmol/L of NaCl, 5 mmol/L of glucose, 1.5 mmol/L of CaCl_2_, 1 mmol/L of magnesium chloride and 0.1% bovine serum albumin (BSA) were used in this assay. Absorbance was determined at 533/617 nm. PI absorption rate (%) = (OD_Sample_−OD_background_)/ (OD_maximum_−OD_background_).
3. Detection of pyroptotic level by PI and Hoechst 33 258 staining: MCNs were stained after intervention of Nigericin and Aβ_1‐42_. After discarding medium, cells were washed with PBS for two times, stained with Hoechst 33 258 staining solution (dilution 1:100, Beyotime Biotechnology Co., Ltd.) for 15 minutes and washed with PBS for two times, followed by observation under microscope, which should display as blue fluorescence for positive cells. In terms of PI staining, 1 μg/mL of PI staining reagent (Beyotime Biotechnology Co., Ltd.) was used for staining for 30 minutes, followed by washing with PBS for two times and observation, which should display as red fluorescence for positive cells. The above two staining methods were used to detect the number of pyroptotic cells.

### 2.5 Detection of GSDMD expression and GSDMD‐mediated signal

1. Immunofluorescence (IF) staining of GSDMD: IF staining of cells was performed. After placing the coverslips in 6‐well plates, MCNs were inoculated to grow into 70% confluency and were induced to undergo pyroptosis. Cells were subsequently fixed with freshly prepared 4% paraformaldehyde (PFA) for 10 minutes, washed with PBS for three times, permeabilized with 0.2% Triton X‐100 for 10 minutes, blocked with 2% BSA for 30 minutes, incubated with anti‐GSDMD monoclonal antibody (dilution 1:300, Abcam) at room temperature for 1 hour, washed with PBS for three times, labelled with IgG antibody (Abcam), stained with 0.5 μg/mL DAPI staining reagent (Solarbio), washed with PBS for two times, sealed and observed under microscope. Since p30‐GSDMD was cleaved N‐terminal p30 protein, it can be labelled with anti‐GSDMD monoclonal antibody.
2. Detection of the relative protein expression by Western blot: MCNs were collected after intervention with Nigericin and Aβ_1‐42_ for 2 hours, washed twice with PBS, lysed with 1.0 mL of pre‐cooled RIPA lysate (Beyotime Biotechnology Co., Ltd.) on ice for 30 minutes and centrifuged at 10 000 *g* for 15 minutes, followed by quantification of the supernatant by BCA kit (Beyotime Biotechnology Co., Ltd.). Protein sample was mixed with 5x loading buffer (20 μL in total), boiled for 8 minutes, subjected to SDS‐PAGE at 80 V and then 120 V and transferred to PVDF membrane at 300 mA for 0.5‐2 hours. The PVDF membranes were blocked with 5% skim milk for 2 hours and incubated with primary antibodies diluted in TBST. Afterwards, the membranes were washed with TBST for two times and incubated with horseradish peroxidase (HRP)‐labelled goat anti‐rabbit secondary antibody (Abcam). Afterwards, ECL method was used, followed by analysis of the optical density using Image Pro‐Plus 6.0 software. GAPDH was used as the loading control. The results were shown as comparison of optical density values between the target protein and the internal control protein. The primary antibodies included anti‐GSDMD and anti‐p30‐GSDMD monoclonal antibodies (dilution 1:500, Abcam); anti‐NLRP3, anti‐pro‐caspase‐1 and anti‐caspase‐1 monoclonal antibodies (dilution 1:800, Abcam); anti‐caspase‐11 monoclonal antibody (dilution 1:50, Abcam). The secondary HRP‐labelled IgG antibody was diluted at 1:1000 (Abcam).

### 2.6 Expression of inflammatory factors in the culture medium

The expression of inflammatory factors in culture medium was examined by ELISA. In brief, MCNs were harvested 2 hours after Nigericin and Aβ_1‐42_ intervention, and the medium was sampled every 30 minutes. After centrifugation at 3000 *g*, the supernatant was collected to determine the levels of inflammatory factors, including IL‐1β, IL‐18 and TNF‐α by ELISA kit (Nanjing Jian Biotechnology Co., Ltd.) according to the manufacturer's instructions. The absorbance was measured at 450 nm using a microplate reader (BioTek), and the result was expressed as pg/mL.

### 2.7 Detection of mouse memory ability

The memory ability of mice was determined by Morris water maze, and video system (Feidi Biotechnology Co., Ltd.) was used. In brief, the water maze consisted of a circular pool, platform and recording system. The pool had a diameter of 120 cm, a height of 40 cm and a water depth of 30 cm. The inner wall of the pool was black, and the temperature was maintained at 20°C. The experiment was performed 1 cm underwater, and the experimental platform was at the midpoint of the four quadrants. Mice were subjected to adaptive training one day before the experiment. Each mouse was trained once, which was allowed to swim from the entrance for 60 seconds. If the mouse could not find the platform, it was artificially guided to stand on the platform for 20 seconds. If the mouse was able to find the platform, then, after standing on the platform for 20 seconds, it was placed back cage. For navigation test, which lasted for seven days, each mouse was examined four times a day (two times in the morning and another two times in the afternoon). The platform was placed in the fourth quadrant. The time interval of entrance with finding and boarding the platform was recorded. If the mouse could not find the platform within 60 seconds, then it was guided to the platform and further stood for 20 seconds. Escape latency was defined as the time interval between entrance into the water and finding the platform. In terms of space exploration experiment, the platform was removed after the navigation test, and mouse was allowed to enter the entrance. Afterwards, investigators recorded the times of crossing the fourth quadrant and the time of staying on the original platform within 60 seconds. Mouse behavioural test was performed at 3, 6, 9, 18 and 27 after AAV9‐siRNA‐caspase‐1 intervention.

### 2.8 Expression of inflammatory cytokines in cerebrospinal fluid and peripheral blood and expression of proteins in cortex of mice

The peripheral blood was extracted from the cerebrospinal fluid and the tail vein of the mice after the behavioural test. The cerebrospinal fluid and peripheral blood were centrifuged to collect the supernatant, followed by detection of inflammatory factors, including IL‐1β and IL‐18 and TNF‐α using ELISA kit. In addition, the expression levels of GSDMD, p30‐GSDMD, caspase‐1, caspase‐11, NLRP3 and pro‐caspase‐1 in mouse cortical tissue were detected by Western blot.

### 2.9 Nissl staining of mouse brain tissue

Paraffin‐embedded mouse brain tissue sections were cut into slices. The slices were treated with xylene dewax (10 minutes x 3), absolute ethanol (2 minutes), 90% ethanol (2 minutes), 70% ethanol (2 minutes) and distilled water (2 minutes). The Nissl staining solution was added to the tissue and stained for 5 minutes. After washing twice with distilled water, the slices were dehydrated with 95% ethanol, treated in xylene for transparent for 5 minutes and sealed with neutral gum, followed by observation under the microscope.

### 2.10 Statistical analysis

All measurement data were shown as $\bar{\chi }\pm s$ , and SPSS 17.0 software was used for statistical analysis and processing. Moreover, t test analysis was used for two independent samples; one‐way ANOVA was used for three or more groups. And LSD method was used for the comparison between the two groups. All of the above tests were two‐sided, and *P* < .05 was considered as statistical significance.

## 3 RESULTS

### 3.1 Aβ_1‐42_ induces pyroptosis MCNs

**FIGURE 1:**
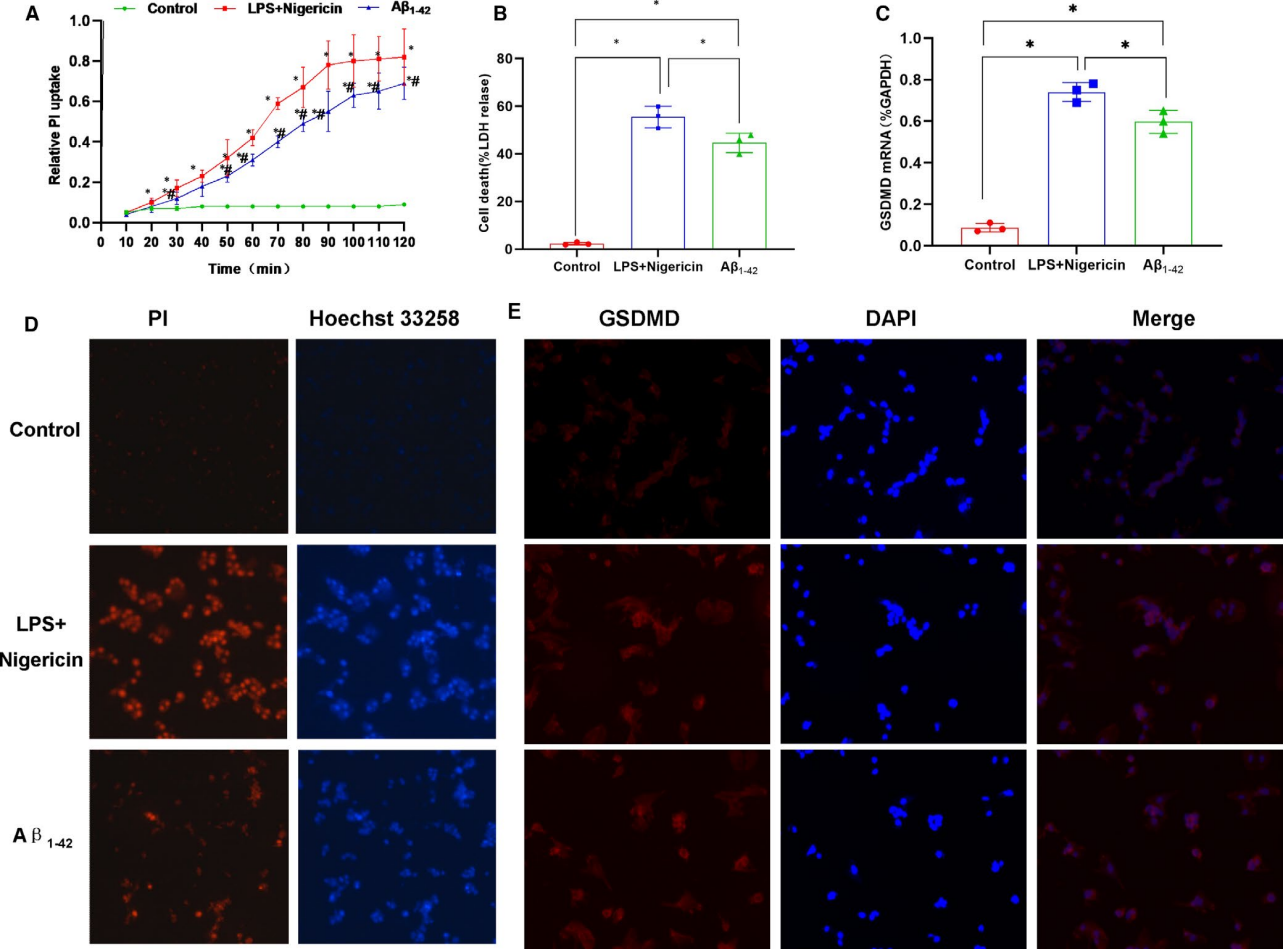
Effects of Aβ_1‐42_ on pyroptosis in MCNs (n = 3). A, Results on relative uptake rate of PI: The relative uptake rate of PI was up‐regulated in the Aβ_1‐42_ group with increasing time, compared with the control group, *P < .05; compared with the LPS + Nigericin group (positive control), *P < .05. Aβ_1‐42_ treatment increased the opening of membrane pores in MCNs. B, Results of LDH on cytotoxicity: Aβ_1‐42_ treatment up‐regulated the release of LDH in MCNs, resulting in cytotoxicity. Comparison between groups, *P < .05. C, Results of GSDMD mRNA expression: Aβ_1‐42_ intervention up‐regulated the mRNA expression of GSDMD, while the mRNA expression of GSDMD was low in the control, indicating that Aβ_1‐42_ promoted mRNA transcription. Comparison between groups, *P < .05. D, Results of PI and Hoechst 33 258 staining in MCNs: The number of positive‐staining cells was relatively less in the control group, while the number of positive‐staining cells in the LPS + Nigericin positive group was significantly increased, indicating the increased number of pyroptotic cells. The number of positive cells was also increased in the Aβ_1‐42_ group, suggesting that Aβ_1‐42_‐induced pyroptosis. E, Results on IF staining of GSDMD: The IF staining of GSDMD was relatively weak in control group, and the IF staining of GSDMD was significantly stronger in LPS + Nigericin and Aβ_1‐42_ groups compared to that in control, indicating the increased expression of GSDMD

**FIGURE 2:**
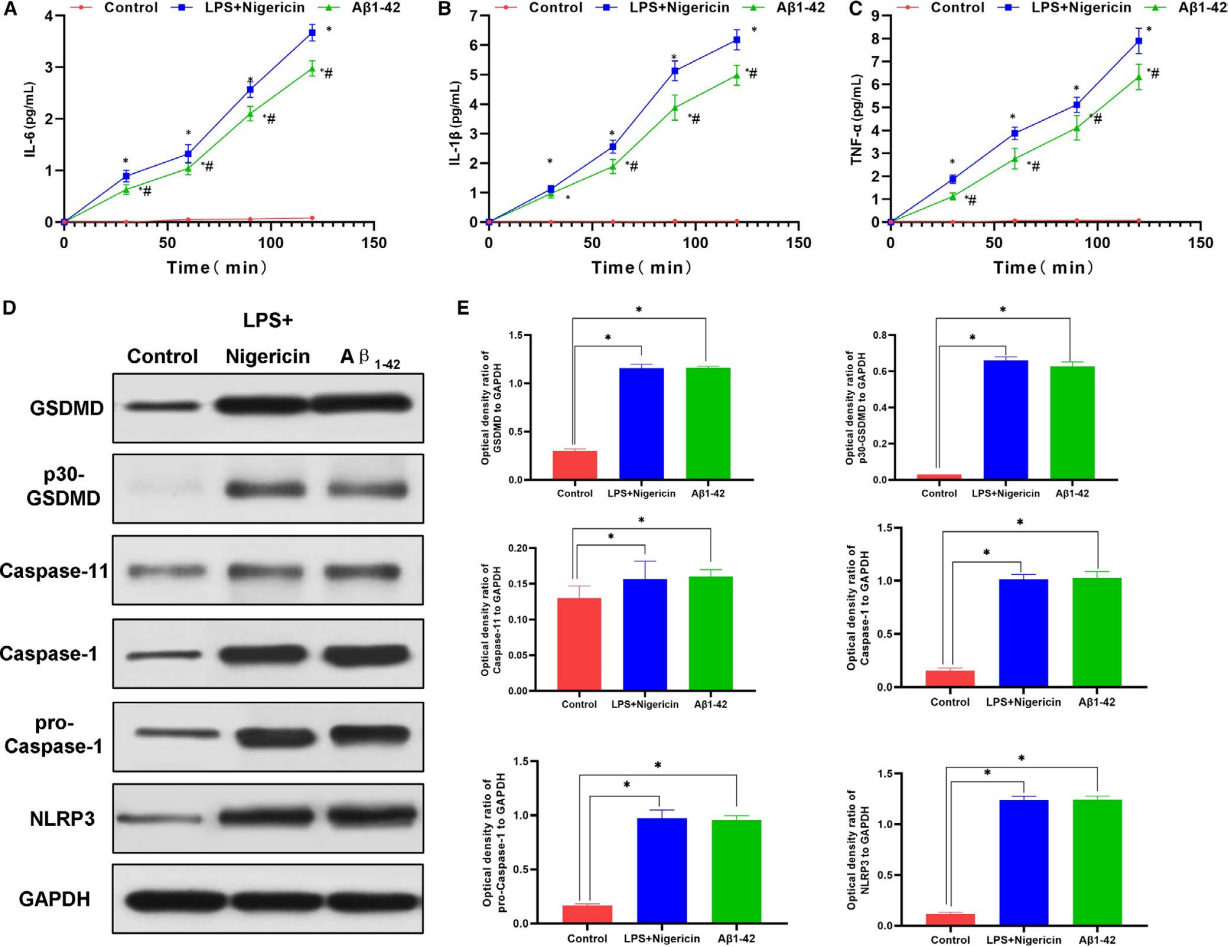
Effects of Aβ1‐42 on inflammatory factor release and expression of pyroptosis‐related proteins in MCNs (n = 3). A‐C, Expression levels of inflammatory factors, including IL‐6, IL‐1β and TNF‐α in cell culture medium. The expression of these inflammatory factors was not significantly changed and remained low over time in the control group, while that was up‐regulated over time in the LPS + Nigericin and Aβ1‐42 groups, indicating increased cell permeability and enhanced release of inflammatory factors. Comparison between Aβ1‐42 group and control group, *P < .05; Comparison with LPS + Nigericin group (positive control), *P < .05. D and E, The expression level of the pyroptosis‐related protein. The expression of GSDMD was relatively low in the control group, p30‐GSDMD was rarely expressed, and the level of cleaved caspase‐1 was also low. After Aβ1‐42 intervention, the expression of GSDMD, the upstream protein NLRP3 and p30‐GSDMD was significantly up‐regulated, while the expression of caspase‐11 was not obvious, indicating that Aβ1‐42 stimulated the expression of caspase‐1 to cleave GSDMD to cause pyroptosis. Comparison between groups, *P < .05

LPS + Nigericin was used to induce pyroptosis as a positive control group. After treatment with Aβ_1‐42_, cells showed obvious swelling, increased permeability and enhanced LDH release rate. Meanwhile, the expression of GSDMD was down‐regulated, the level of cleaved p30‐GSDMD was up‐regulated, and the expression of GSDMD‐cleaved caspase‐1 and upstream protein NLRP3 of GSDMD was up‐regulated, while the expression of caspase‐11 was not significantly changed. ELISA assay results showed that the levels of inflammatory factors, including IL‐1β, IL‐18 and TNF‐α, were up‐regulated in the culture medium, which was significantly different from those in the control group (*P* < .05). In addition, Aβ_1‐42_ could induce pyroptosis, which might be mediated by NLRP3‐caspase‐1 signalling. GSDMD played an important role in pyroptosis (shown in Figures 1 and 2).

### 3.2 Effects of caspase‐1 or GSDMD silencing on Aβ_1‐42_‐induced pyroptosis in MCNs

**FIGURE 3:**
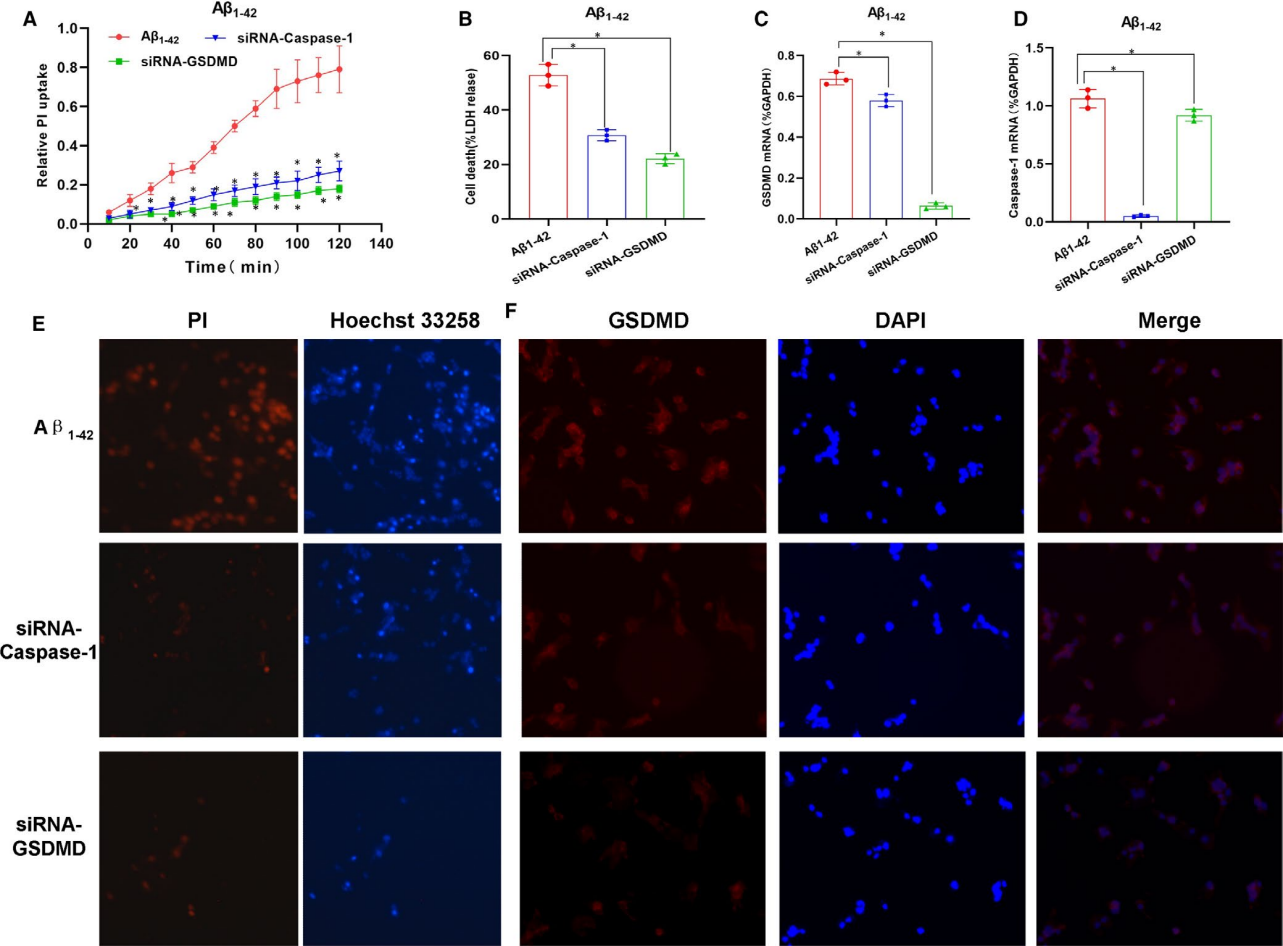
caspase‐1 or GSDMD silencing inhibited Aβ_1‐42_‐induced pyroptosis in MCNs (n = 3). A, Results on the relative uptake rate of PI. The relative uptake rate of PI was significantly increased in Aβ_1‐42_ intervention group, while siRNA‐caspase‐1 and siRNA‐GSDMD could significantly inhibit the relative uptake rate of PI, indicating that inhibition of caspase‐1 or GSDMD suppressed the opening of cell membranes and decreased PI uptake, compared to Aβ_1‐42_, *P < .05. B, Results on LDH cytotoxicity: Aβ_1‐42_ intervention up‐regulated LDH release in MCNs, causing cytotoxicity. The cytotoxicity levels were down‐regulated in siRNA‐caspase‐1 and siRNA‐GSDMD groups, compared between groups, *P < .05. C and D, The mRNA expression of GSDMD and caspase‐1: Aβ_1‐42_ intervention up‐regulated the expression of GSDMD and caspase‐1, while siRNA intervention down‐regulated the mRNA expression of GSDMD and caspase‐1. Comparison between groups, *P < .05. E, Results of PI and Hoechst 33 258 staining in MCNs: Aβ_1‐42_ intervention caused relatively more positive‐staining cells, while GSDMD and caspase‐1 silencing resulted in significantly decreased number of positive cells, indicating that inhibition of caspase‐1 or GSDMD could decrease the opening level of the membrane pores. F, Results of GSDMD IF staining: After Aβ_1‐42_ intervention, the GDXMD IF staining was relatively strong, indicating the high expression of GSDMD, while the levels of GSDMD were significantly decreased in siRNA‐caspase‐1 and siRNA‐GSDMD groups, suggesting that inhibition of caspase‐1 or GSDMD decreased the expression of GSDMD

**FIGURE 4:**
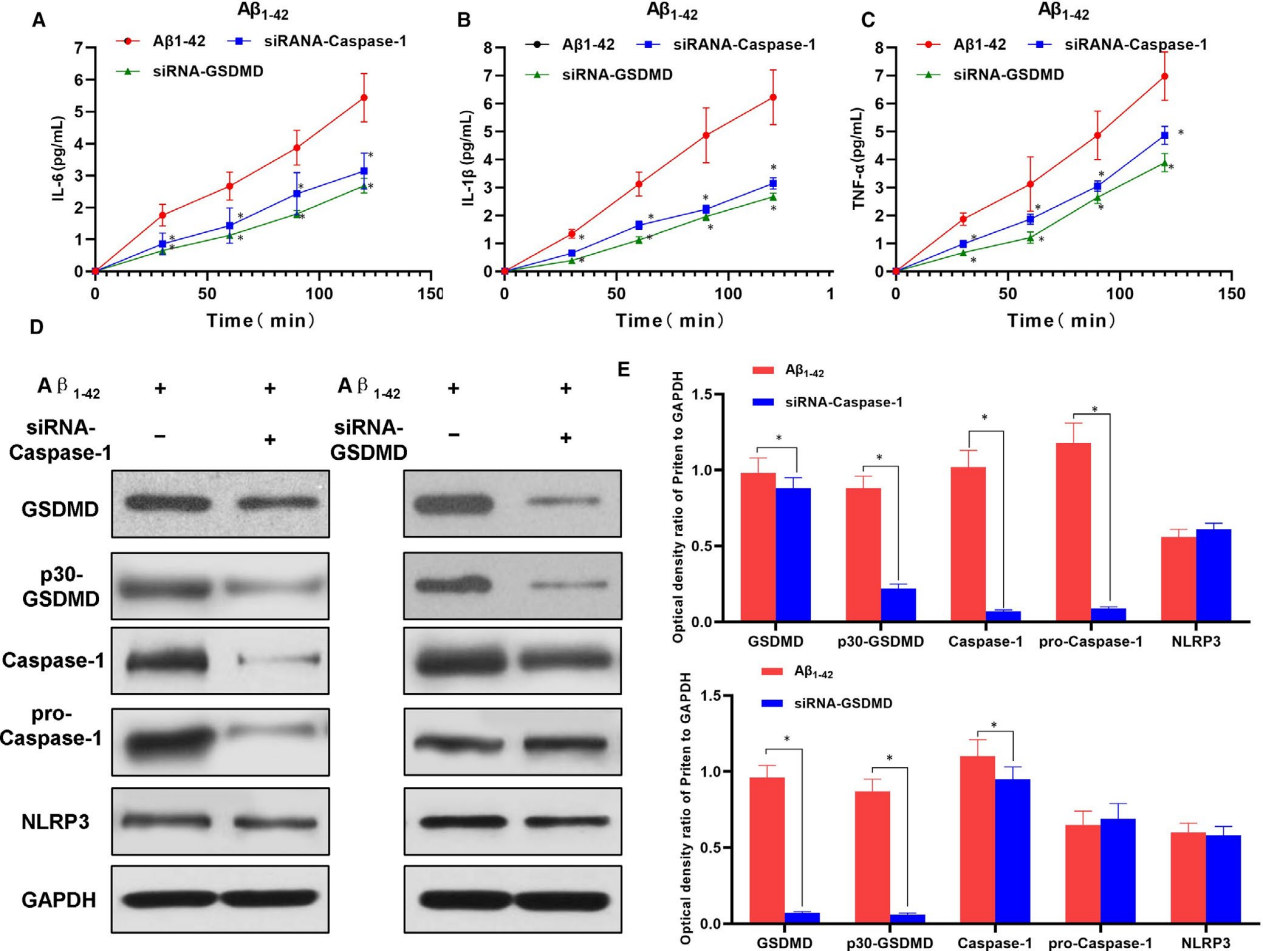
Effects of caspase‐1 or GSDMD silencing on Aβ_1‐42_‐induced inflammatory factor release and expression of pyroptosis‐related protein in MCNs (n = 3). A‐C, The expression levels of inflammatory factors, including IL‐6, IL‐1β and TNF‐α in the culture medium. The expression of inflammatory factors was increased over time in the Aβ_1‐42_ group, indicating increase cell permeability and the release of inflammatory factors. However, the levels of inflammatory factor were significantly down‐regulated in siRNA‐caspase‐1 and siRNA‐GSDMD groups, compared with Aβ1‐42 group, *P < .05. D and E, The expression of pyroptosis‐related proteins. The expression levels of GSDMD and p30‐GSDMD were higher in the Aβ_1‐42_ group. After caspase‐1 inhibition, the level of p30‐GSDMD was down‐regulated, while the expression of GSDMD and NLRP3 was not significantly changed. After suppression of GSDMD, the levels of GSDMD and p30‐GSDMD were lower, but did not affect the expression of upstream cleaved proteins caspase‐1 and NLRP3. Comparison between groups, *P < .05

The expression of caspase‐1 was silenced by siRNA, followed by intervention with 25 μmol/L Aβ_1‐42_. As a result, the cell permeability was increased, the expression of inflammatory factors in the medium was up‐regulated, the positive rate of PI staining was up‐regulated, and the release rate of LDH was enhanced in the Aβ_1‐42_ group. Compared with the Aβ_1‐42_ group, the membrane permeability was down‐regulated, the level of inflammatory factors in the culture medium was down‐regulated, and the level of key protein p30‐GSDMD was down‐regulated in siRNA‐caspase‐1 group, indicating that silencing caspase‐1 could inhibit the cleavage of GSDMD protein and simultaneously inhibit pyroptosis and the release of inflammatory factors. Similarly, after GSDMD silencing using siRNA and subsequent Aβ_1‐42_ intervention, the cell permeability was significantly decreased in the siRNA‐GSDMD group than that in the Aβ_1‐42_ group, and the expression of inflammatory factors in the culture medium was down‐regulated, which, however, did not affect the expression of upstream signal NLRP3 and caspase‐1. The above assays suggested that silencing caspase‐1 could inhibit Aβ_1‐42_‐induced pyroptosis, and similarly, inhibition of downstream executive protein GSDMD could also suppress pyroptosis, indicating that caspase‐1‐mediated GSDMD cleavage was the main signal in Aβ_1‐42_‐induced pyroptosis in nerve cells (shown in Figures 3 and 4).

### 3.3 Effects of p30‐GSDMD oligomerization inhibitor on Aβ_1‐42_‐induced pyroptosis in MCNs

**FIGURE 5:**
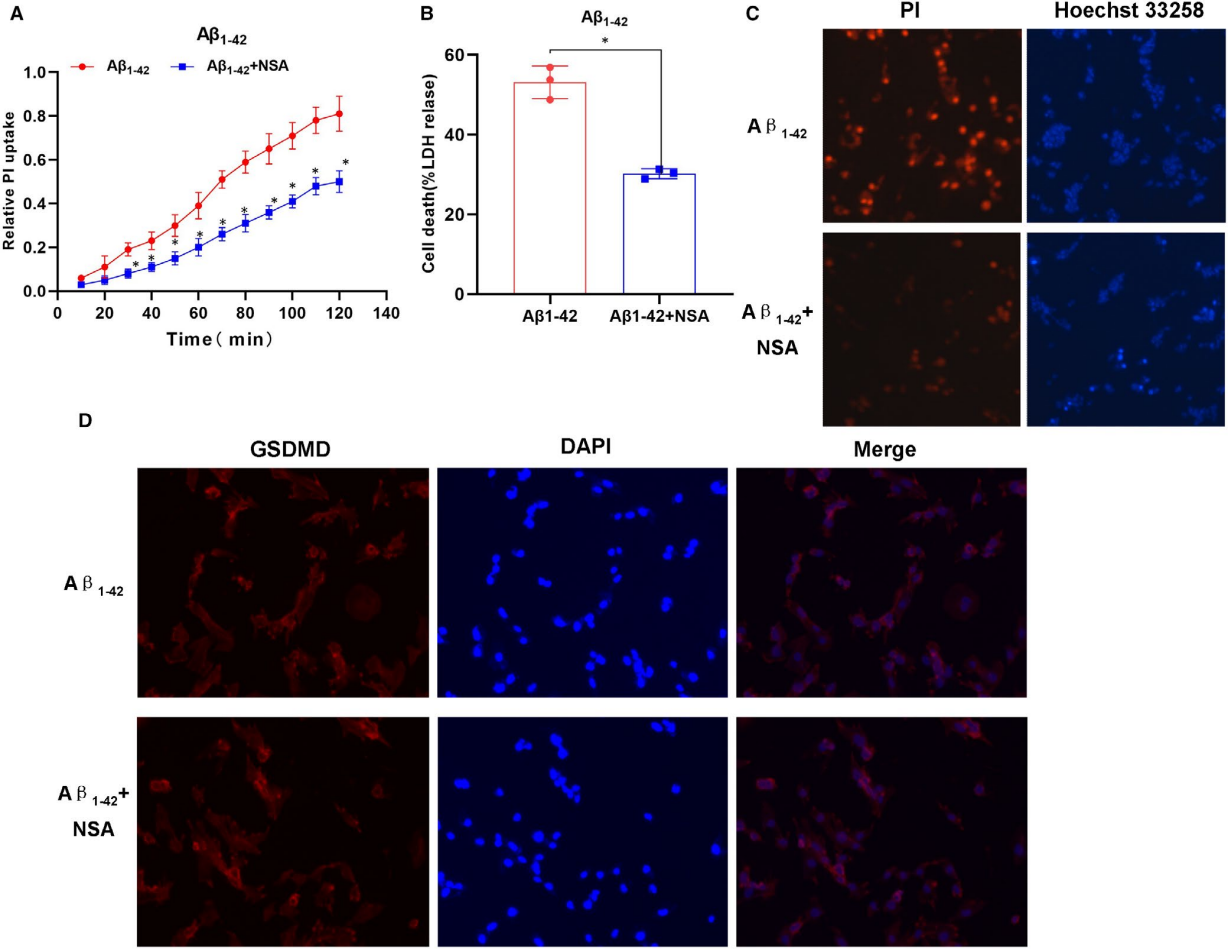
p30‐GSDMD oligomerization inhibitor NSA inhibited Aβ_1‐42_‐induced pyroptosis in MCNs (n = 3). A, Results of the relative uptake rate of PI: Aβ_1‐42_ treatment could induce increased relative uptake rate of PI in MCNs, and NSA intervention could inhibit the opening of cell membrane and decrease PI uptake, compared with Aβ_1‐42_, *P < .05. B, Results of LDH cytotoxicity: Aβ_1‐42_ treatment up‐regulated the release of LDH in MCNs, causing cytotoxicity. After NSA intervention, the cytotoxicity level was down‐regulated. Comparison between groups, *P < .05. C, Results of PI and Hoechst 33 258 staining: Aβ_1‐42_ intervention increased the number of positive‐staining cells, while NSA intervention significantly decreased the number of positive‐staining cells, and NSA could attenuated the opening degree of membrane pores. D, Results of GSDMD IF staining: Aβ_1‐42_ intervention caused relatively stronger IF staining of GDXMD, indicating the relatively higher expression of GSDMD; however, the level of GSDMD was not significantly changed after NSA intervention, suggesting that NSA did not affect the expression of GSDMD, only inhibited oligomerization of p30‐GSDMD

**FIGURE 6:**
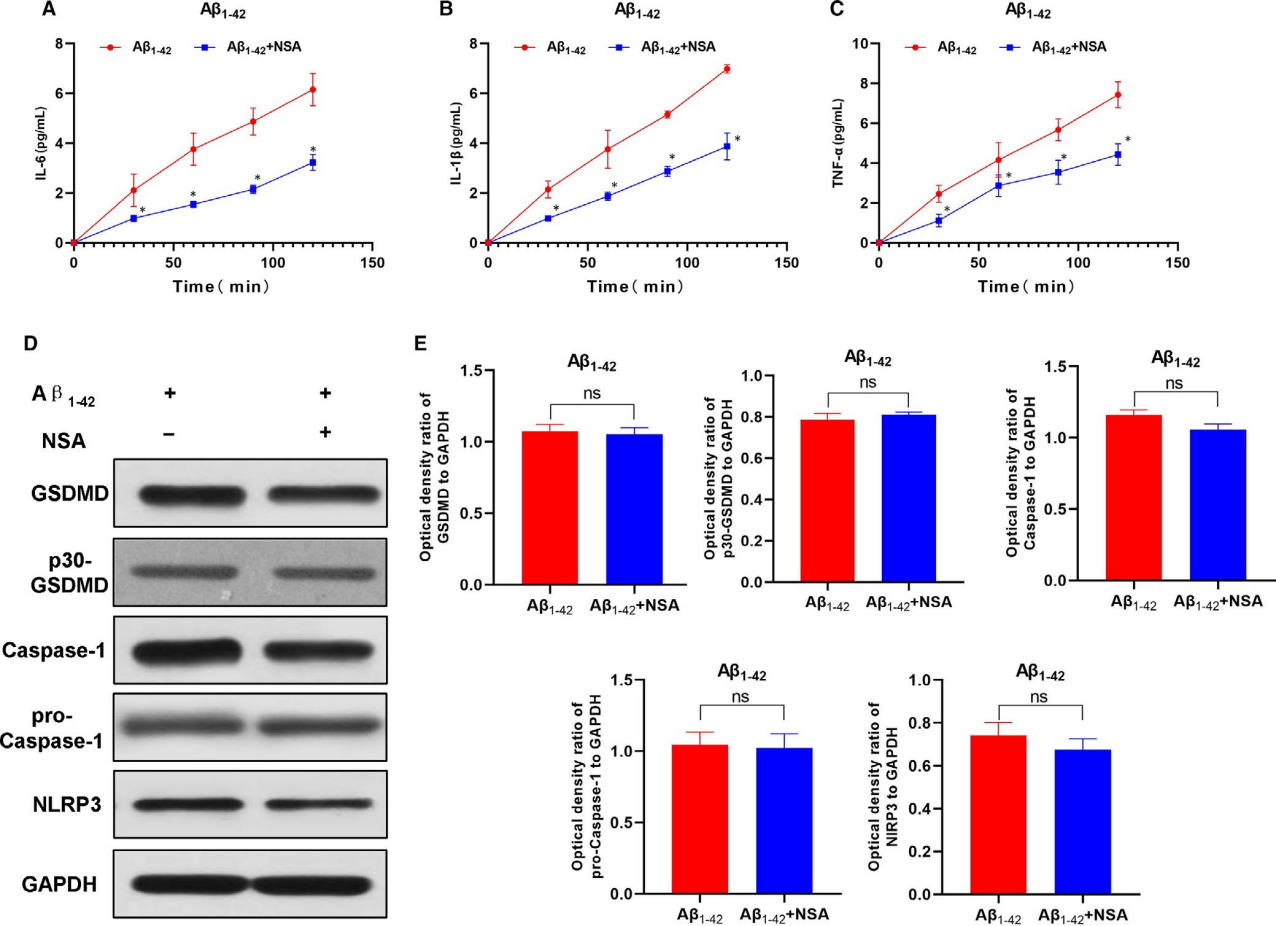
p30‐GSDMD oligomerization inhibitor NSA inhibited Aβ_1‐42_‐induced inflammatory factor release and expression of pyroptotic protein (n = 3). A‐C, The expression levels of inflammatory factors, including IL‐6, IL‐1β and TNF‐α in cell culture medium. The expression of these inflammatory factors was increased over time in the Aβ_1‐42_ group, indicating the increased cell permeability and the release of inflammatory factors. And the levels of inflammatory factors were significantly down‐regulated after NSA intervention, compared with Aβ_1‐42_ group, *P < .05. D and E, The expression level of pyroptosis‐related proteins. The expression levels of GSDMD and p30‐GSDMD (executive protein of pyroptosis) were relatively higher in the Aβ_1‐42_ group; however, the levels of GSDMD and p30‐GSDMD were not significantly changed after NSA intervention. Meanwhile, the expression of caspase‐1 (cleavage protein) or upstream NLRP3 was not significantly changed. Comparison between groups, ^ns^P > 0.05. These results indicated that NSA did not affect the expression of pyroptosis signalling protein nor did it affect the cleavage, but affected the oligomerization of the effector protein p30‐GSDMD and suppressed the oligomerization of p30‐GSDMD to open the membrane pore

p30‐GSDMD was anchored to the cell membrane after oligomerization to open the cell pores. NSA was an oligomerization inhibitor, and inhibition of p30‐GSDMD oligomerization could inhibit pyroptosis and the release of inflammatory factors. In our study, pre‐treatment with NSA could significantly inhibit Aβ_1‐42_‐induced pyroptosis in MCNs, down‐regulated the opening degree of cell membrane pores and decreased the release rate of LDH. However, NSA did not affect the expression of GSDMD and p30‐GSDMD nor did it affect the expression of upstream NLRP3 and caspase‐1, while it could down‐regulate the release of inflammatory factors and decrease the levels of IL‐1β, IL‐18 and TNF‐α in the medium. These results indicated that NSA inhibited the opening of membrane pores, but did not affect the expression of upstream proteins and the cleavage of GSDMD (shown in Figures 5 and 6).

### 3.4 Relieving effects and mechanism of caspase‐1 inhibition on behaviour in APP/PS1 mice

**FIGURE 7:**
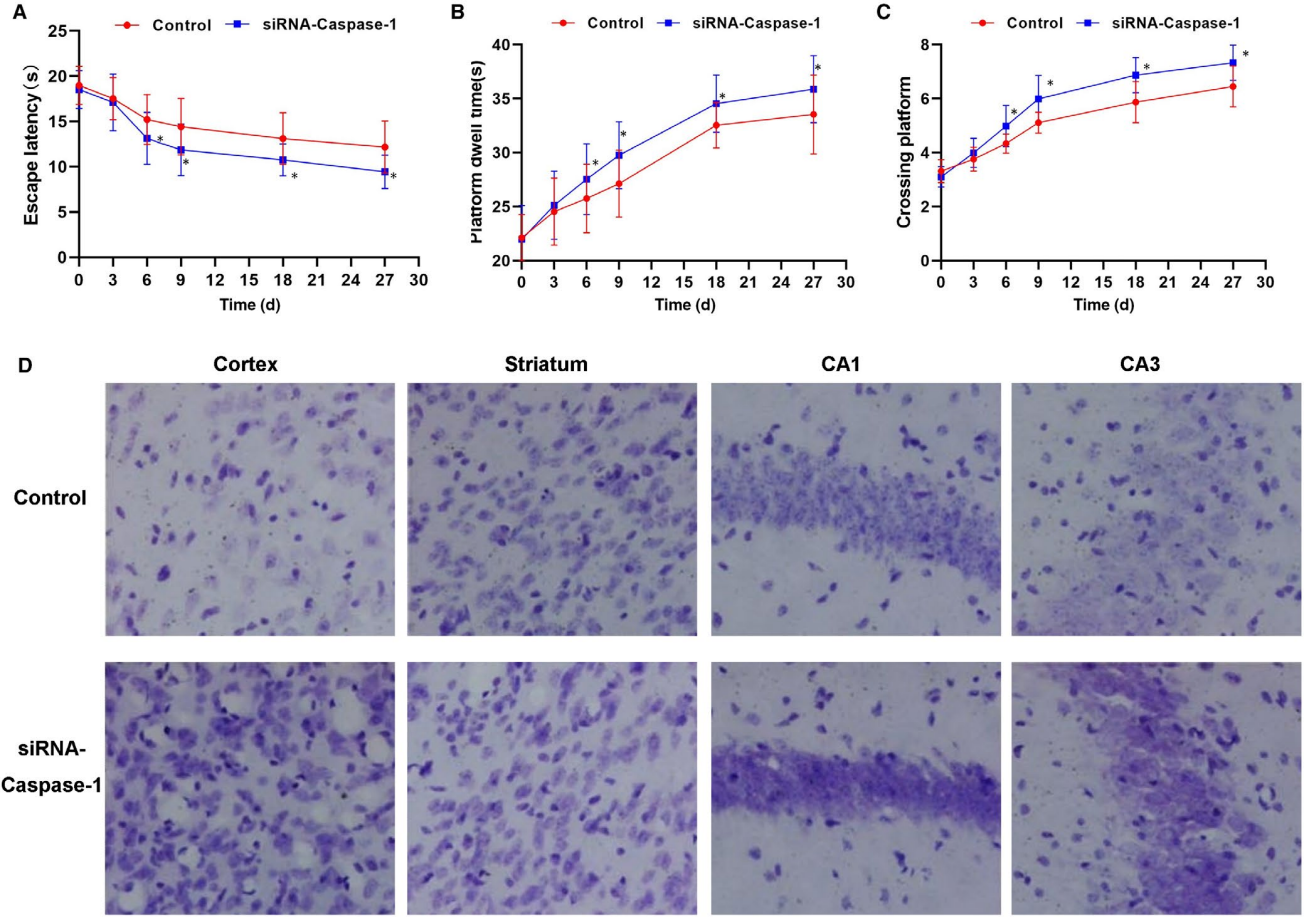
Inhibition of caspase‐1 attenuated cognitive and neuronal damage in AD APP/PS1 mice (n = 3). A, Results of escape latency of mice: The escape latency was relatively longer in the control group. While the escape latency was significantly shorter in the siRNA‐caspase‐1 group than the control group since the 6th day, indicating that caspase‐1 inhibition could improve the cognitive ability of mice, compared with control group, *P < .05. B, Results of staying time on platform: The staying time on platform was significantly longer in the siRNA‐caspase‐1 group than the control group since the 6th day, compared with the control group, *P < .05. C, Results of number of platform crossing: The number of platform crossing was significantly higher in the siRNA‐caspase‐1 group than the control group since the 6th day, compared with the control group, *P < .05. D, Nissl staining of mouse brain tissue: the staining of neurons was light in the hippocampus, cortex and striatum in the control group, with less positive cells, especially in the cortex and hippocampus. The nerve cells were significantly less in the control group than the siRNA‐caspase‐1 group. The staining of nerve cells was dense in the siRNA‐caspase‐1 group

**FIGURE 8:**
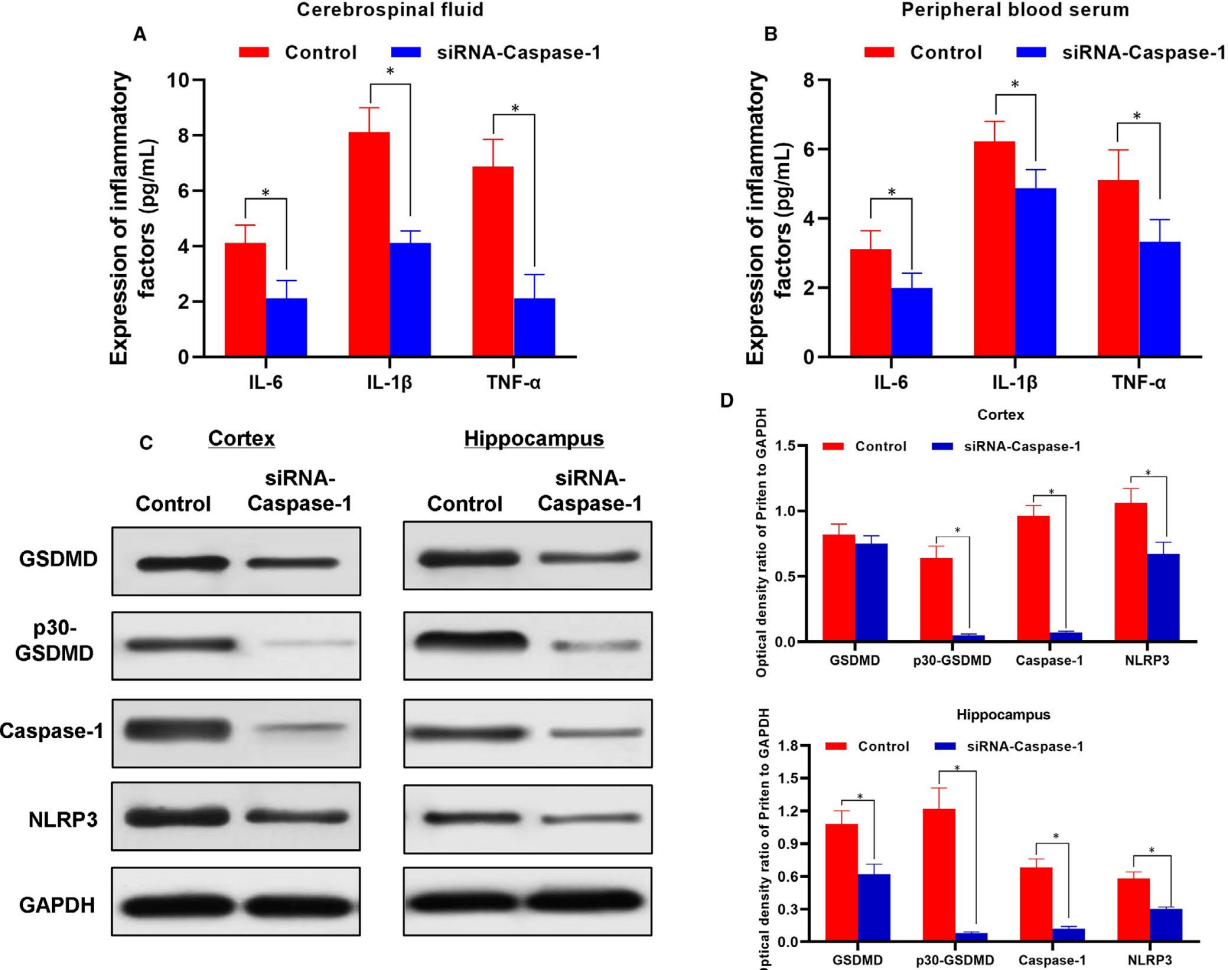
Effects of caspase‐1 inhibition on inflammatory factor release and pyroptotic protein in AD mice (n = 3). A, The expression of inflammatory factors in cerebrospinal fluid of mice. The expression levels of IL‐6, IL‐1β and TNF‐α in cerebrospinal fluid in the control group were significantly lower than those in the siRNA‐caspase‐1 group. Comparison between groups, *P < .05. B: Expression of inflammatory cytokines in peripheral blood of mice. The expression levels of IL‐6, IL‐1β and TNF‐α in peripheral blood in the control group were significantly lower than those in the siRNA‐caspase‐1 group. Comparison between groups, *P < .05. B‐D, Expression of pyroptotic protein in mouse cortex and hippocampus. The expression of p30‐GSDMD and caspase‐1 in the cortex and hippocampus of the siRNA‐caspase‐1 group was significantly lower than that of control group. The expression of upstream protein, NLRP3 and GSDMD was slightly lower than the control group. These results showed that siRNA‐caspase‐1 significantly inhibited the cleavage of GSDMD, while had little effect on the expression of the upstream protein NLRP3. Comparison between groups, *P < .05

APP/PS1 mice were treated with AAV9‐siRNA‐caspase‐1, and the expression of caspase‐1 in whole brain was inhibited. Mice in the control group were not treated. As a result, mice in the control group showed significant cognitive impairment, and the cognitive ability of mice was significantly different between the siRNA‐caspase‐1 group and the control group. First, the escaping latency of mice in the siRNA‐caspase‐1 group was significantly shorter than that of the control group, which became significant from the 6th day. The staying time on platform and the number of crossing platforms were significantly higher in mice in the siRNA‐caspase‐1 group than that of the control group, indicating the memory capacity of mice was improved. Nissl staining also showed that the neuronal damage in the hippocampus, cortex and striatum was significantly less in the siRNA‐caspase‐1 group than that in the control group. The staining was light with sparse nerve cells in the control group, while the staining was dense with significantly better distribution of nerve cells in the siRNA‐caspase‐1 group. The expression of inflammatory factors from cerebrospinal fluid and peripheral blood of mice showed the levels of IL‐6, IL‐1β and TNF‐α were lower in the siRNA‐caspase‐1 group than those in the control group. The levels of caspase‐1 and p30‐GSDMD in mouse cortex and hippocampus were significantly decreased, and the level of GSDMD was also down‐regulated, indicating that siRNA‐caspase‐1 could inhibit the cleavage of GSDMD in brain tissue and inhibit the release of inflammatory factors. Animal experiments showed that siRNA‐caspase‐1 could improve the cognitive ability of PD mice, which was related to the inhibition of inflammatory factor release (shown in Figures 7 and 8).

## 4 DISCUSSION

Alzheimer's disease is a degenerative disease of the central nervous system characterized by progressive cognitive impairment and behavioural impairment.^15^, ^16^ The β‐amyloid cascade theory is the centre of the pathogenesis of AD: Aβ can cause oxidative stress, axonal damage and synaptic loss, eventually leading to the death of nerve cells.^17^, ^18^ In the pathogenesis of AD, chronic neuroinflammatory response is also an important pathophysiological characteristic. Previous studies have indicated that Aβ can activate glial cells, especially microglia and astrocytes to produce corresponding inflammatory factors, thereby promoting the progression of AD.^19^ However, the study of neuroinflammation is mainly concentrated in Aβ‐induced glial cells; however, whether Aβ promotes the autoinflammatory response of nerve cells is rarely reported, and there are few studies on mechanism. Nevertheless, the neuronal damage of Aβ is clear and direct; therefore, we speculate that Aβ may have a mechanism that directly induces inflammatory factors release from nerve cells.

Pyroptosis is a new type of cell death, mainly characterized by the opening of the cell membrane pores, the swelling of cells and the release of a large amount of cellular contents, thereby leading to cell rupture.^20^, ^21^ The key executive protein is GSDMD, which is a member of the Gasdermin protein family. Although GSDME has also been reported to be able to mediate pyroptosis, GSDMD is the most important one, with relatively thorough investigations.^22^, ^23^ In the early stage of pyroptosis, the upstream signal is activated, manifested as the activation of the NLRP3 inflammasome and recruitment of NLRP3 inflammasome to small bodies to subsequently cleave pro‐caspase‐1 to form mature caspase‐1.^24^ GSDMD is formed under the cleavage of caspase‐1. GSDMD forms a p30 terminal after cleavage, which is an easy oligomerization protein. p30 oligomerization can be localized on the cell membrane, to open the membrane pore channel and to release a multiple inflammatory factors to the extracellular space.^25^ In addition, NLRP3 inflammasome can also serve as a cleavage platform for inflammatory factors such as IL‐1β, and pro‐IL‐1β is cleaved by NLRP3 to form mature IL‐1β.^26^ Therefore, NLRP3 is the initiating protein for pyroptosis. caspase‐1 is an important cleaving protein, and p30‐GSDMD is the essential protein for pyroptosis. Anyhow, GSDMD cleavage and p30 formation truly trigger pyroptosis.

In this study, we found that Aβ_1‐42_ can directly act on MCNs to induce pyroptosis, to open cell membrane pores, to significantly increase the PI uptake level and to enhance the number of pyroptotic cells. In addition, the increased expression of GSDMD and p30‐GSDMD and the enhanced levels of upstream NLRP3 protein and cleaved caspase‐1 play critical roles, indicating that Aβ_1‐42_ regulates pyroptosis by NLRP3‐caspase‐1 signalling. caspase‐11 has also been reported to cleave GSDMD; thus, we also detected the expression of caspase‐11. As a result, the expression of caspase‐11 after Aβ_1‐42_ intervention was not obviously changed. Therefore, we focused on the canonical pyroptosis mediated by caspase‐1. caspase‐1 and GSDMD silencing by siRNA could both significantly suppress pyroptosis, and the expression of p30‐GSDMD was down‐regulated, which further confirming that caspase‐1‐GSDMD is the main signal of Aβ_1‐42_‐induced pyroptosis. NSA is a p30‐GSDMD oligomerization inhibitor,^27^ which mainly inhibits the oligomerization of p30‐GSDMD. In this study, MCNs were pre‐treated with NSA. Consequently, NSA pre‐treatment suppressed Aβ_1‐42_‐induced MCN pyroptosis, mainly manifested by inhibition of cell membrane permeability and suppression of inflammatory factor release, but not affecting the expression of key proteins such as NLRP3 and GSDMD, caspase‐1. This assay demonstrates that inhibition of p30‐GSDMD oligomerization can inhibit the opening of membrane pores, validating that p30‐GSDMD oligomerization is one of the key steps in MCN pyroptosis. Therefore, we have fully demonstrated that caspase‐1‐mediated GSDMD cleavage is the main mechanism of Aβ_1‐42_‐induced neuronal pyroptosis at the cellular level.

To further investigate the role of neuronal pyroptosis in AD, AAV9‐packaged siRNA‐caspase‐1 was used to interfere with the expression of caspase‐1 in the brain tissue of APP/PS1 mice. The behavioural results of animal cognitive ability showed that caspase‐1 inhibition could relieve the cognitive ability of mice, attenuate the degree of nerve cell damage was reduced in brain tissue (hippocampus, striatum and cortex) and simultaneously decrease the levels of inflammatory factors in cerebrospinal fluid and peripheral blood, indicating that siRNA‐caspase‐1 inhibits the pyroptosis of nerve cells and attenuates nerve damage. Inhibition of pyroptosis can relieve the cognitive ability of AD. Previous reports of neuroinflammation were all aimed at glial cells, including microglia, astrocytes and so on. However, there are few studies on the pyroptosis of nerve cells. This study focuses on Aβ‐induced pyroptosis of nerve cells. Different from previous studies, previous studies mainly focused on apoptosis of nerve cells, rather than inflammatory death.

In summary, in the present study, we find that Aβ_1‐42_ can directly induce neuronal pyroptosis through the canonical pyroptotic signal caspase‐1‐GSDMD. Pyroptosis intervention could attenuate neuroinflammation of AD, reduce nerve cell damage and improve AD cognitive impairment. Our present findings are considered as a new mechanism of Aβ_1‐42_‐induced nerve damage and neuroinflammation, which provides new references for AD research. Thus, neuroinflammation not only is limited to glial cells, but also directly occur within nerve cells.

## 5 CONCLUSION

In this study, we main research the mechanism of Aβ_1‐42_ reduced pyroptosis in neurons. We found that Aβ_1‐42_ could induce pyroptosis by GSDMD protein, and NLRP3‐caspase‐1 signalling was an important signal to mediate GSDMD cleavage, which plays an important role in Aβ_1‐42_‐induced pyroptosis in neurons. Therefore, GSDMD is expected to be a novel therapeutic target for AD.

## CONFLICT OF INTEREST

No Competing interests.

Consent for publication: All authors’ approval published the article.

## AUTHOR CONTRIBUTION

**chenyang han:** Investigation (equal). **yi yang:** Investigation (equal); Supervision (equal). **qiaobing guan:** Data curation (equal). **heping shen:** Data curation (equal); Methodology (equal); Project administration (equal). **xiaoling zhang:** Data curation (equal); Resources (equal); Writing‐original draft (equal). **yongjia sheng:** Methodology (equal); Project administration (equal); Writing‐review & editing (equal). **jin wang:** Methodology (equal); Project administration (equal); Writing‐review & editing (equal). **xiaohong zhou:** Methodology (equal); Project administration (equal); Validation (equal); Writing‐review & editing (equal). **wenyan Li:** Investigation (equal); Resources (equal); Validation (equal); Writing‐review & editing (equal). **li guo:** Conceptualization (equal); Formal analysis (equal); Funding acquisition (equal). **qingcai jiao:** Conceptualization (equal); Funding acquisition (equal); Supervision (equal); Validation (equal).

## ETHICAL APPROVAL

The study approved with Ethics Committee.

## Data Availability

The data and material were availability.
