# Corrigendum

**DOI:** 10.1111/jcmm.17134

**Published:** 2022-01-08

In Chenyang Han et al.^1^, several incorrect images were used in the published version. They include: PI staining in LPS + Nigericin panel in Figure 1D, Caspase‐11 gel panel in Figure 2D, Caspase‐11 and GAPDH gel panel in Figure 4D, and GAPDH and NLRP3 gel panel in Figure 6D. The correct figures are shown below. The authors confirm all results and conclusions of this article remain unchanged.

**FIGURE 1:**
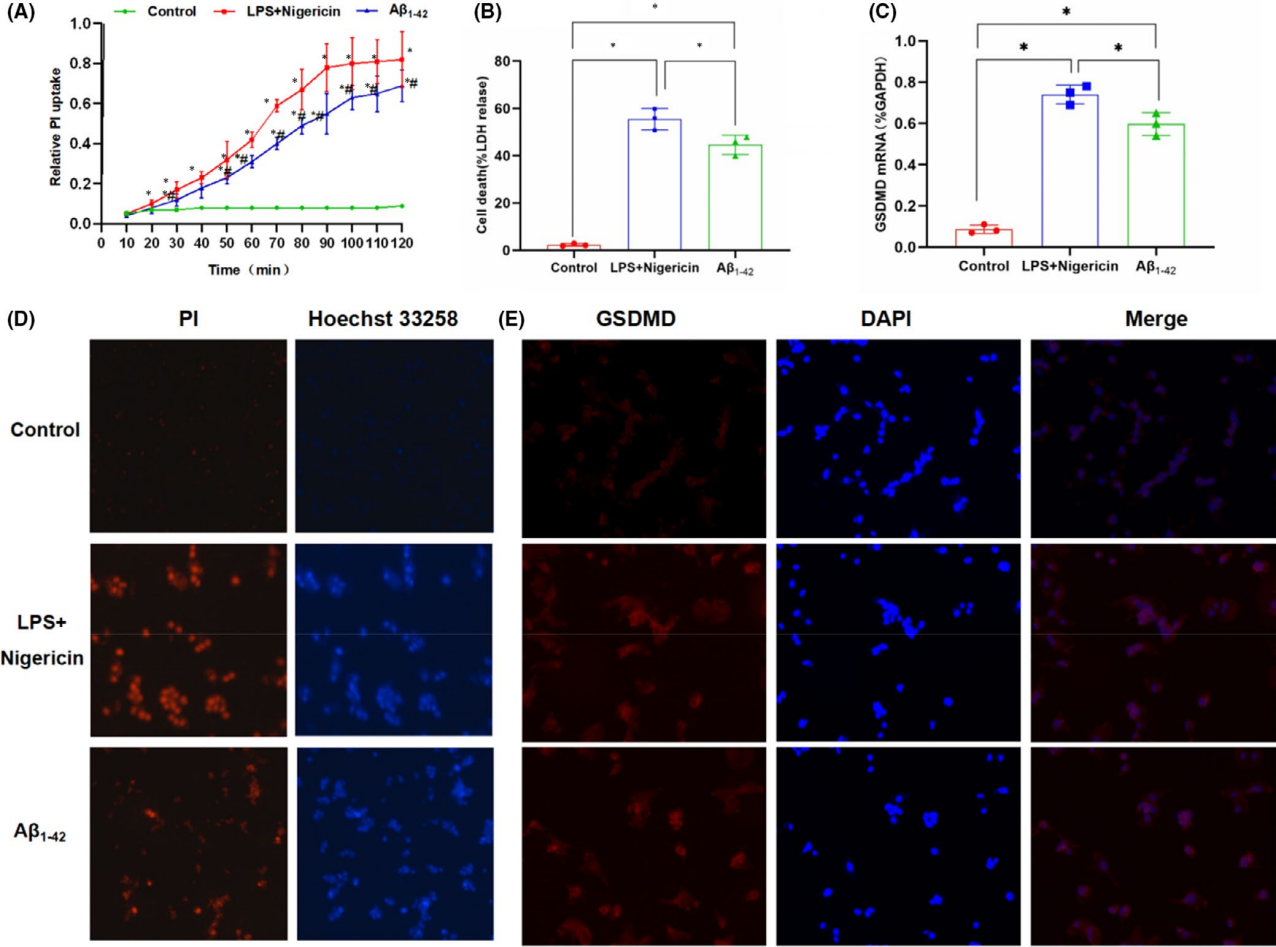
Effects of Aβ_1‐42_ on pyroptosis in MCNs (n = 3). A, Results on relative uptake rate of PI: The relative uptake rate of PI was up‐regulated in the Aβ_1‐42_ group with increasing time, compared with the control group, *P < .05; compared with the LPS + Nigericin group (positive control), *P < .05. Aβ_1‐42_ treatment increased the opening of membrane pores in MCNs. B, Results of LDH on cytotoxicity: Aβ_1‐42_ treatment up‐regulated the release of LDH in MCNs, resulting in cytotoxicity. Comparison between groups, *P < .05. C, Results of GSDMD mRNA expression: Aβ_1‐42_ intervention up‐regulated the mRNA expression of GSDMD, while the mRNA expression of GSDMD was low in the control, indicating that Aβ_1‐42_ promoted mRNA transcription. Comparison between groups, *P < .05. D, Results of PI and Hoechst 33,258 staining in MCNs: The number of positive‐staining cells was relatively less in the control group, while the number of positive‐staining cells in the LPS + Nigericin positive group was significantly increased, indicating the increased number of pyroptotic cells. The number of positive cells was also increased in the Aβ_1‐42_ group, suggesting that Aβ_1‐42_‐induced pyroptosis. E, Results on IF staining of GSDMD: The IF staining of GSDMD was relatively weak in control group, and the IF staining of GSDMD was significantly stronger in LPS + Nigericin and Aβ_1‐42_ groups compared to that in control, indicating the increased expression of GSDMD

**FIGURE 2:**
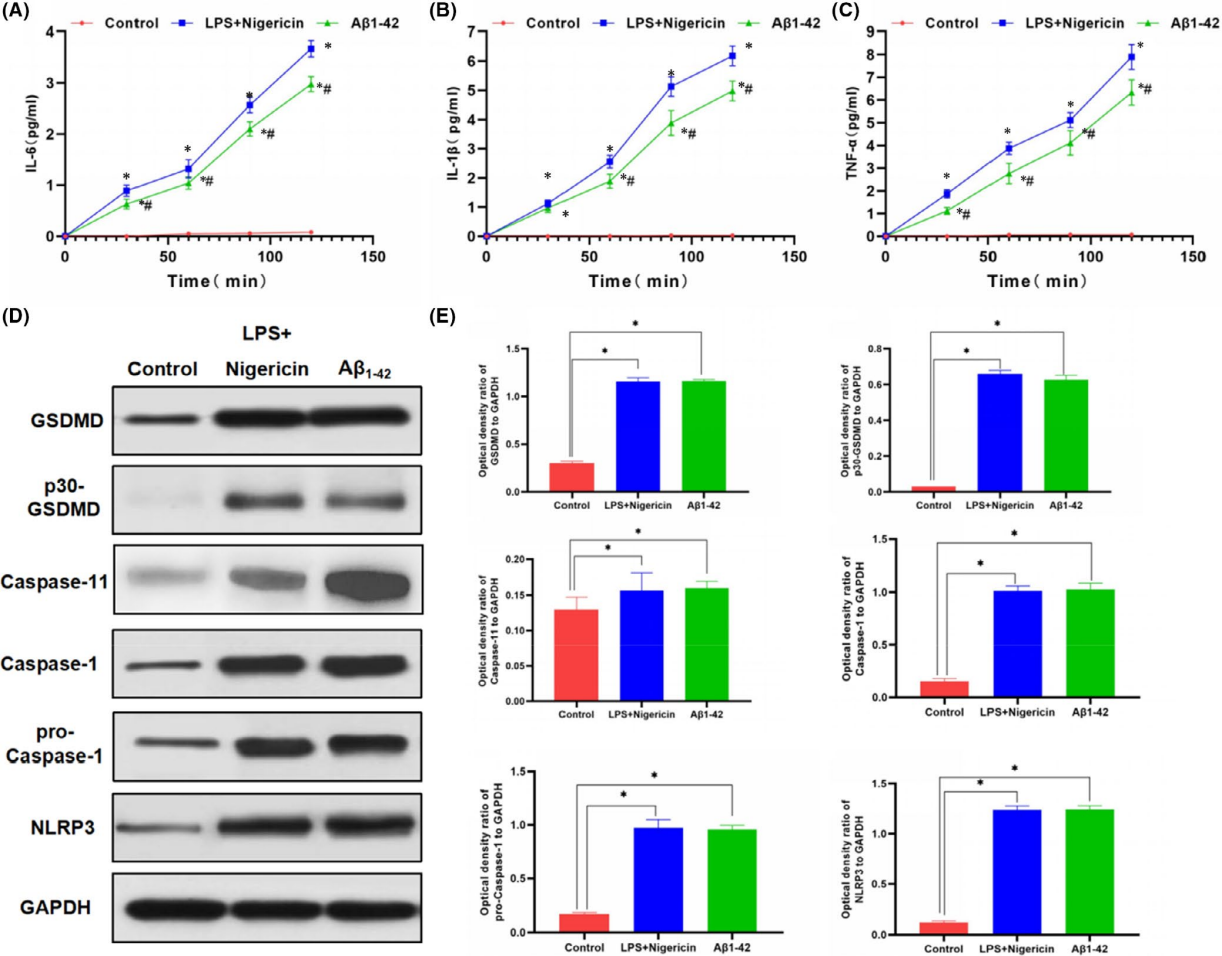
Effects of Aβ1‐42 on inflammatory factor release and expression of pyroptosis‐related proteins in MCNs (n = 3). A‐C, Expression levels of inflammatory factors, including IL‐6, IL‐1β and TNF‐α in cell culture medium. The expression of these inflammatory factors was not significantly changed and remained low over time in the control group, while that was up‐regulated over time in the LPS + Nigericin and Aβ1‐42 groups, indicating increased cell permeability and enhanced release of inflammatory factors. Comparison between Aβ1‐42 group and control group, *P < .05; Comparison with LPS + Nigericin group (positive control), *P < .05. D and E, The expression level of the pyroptosis‐related protein. The expression of GSDMD was relatively low in the control group, p30‐GSDMD was rarely expressed, and the level of cleaved caspase‐1 was also low. After Aβ1‐42 intervention, the expression of GSDMD, the upstream protein NLRP3 and p30‐GSDMD was significantly up‐regulated, while the expression of caspase‐11 was not obvious, indicating that Aβ1‐42 stimulated the expression of caspase‐1 to cleave GSDMD to cause pyroptosis. Comparison between groups, *P < .05

**FIGURE 4:**
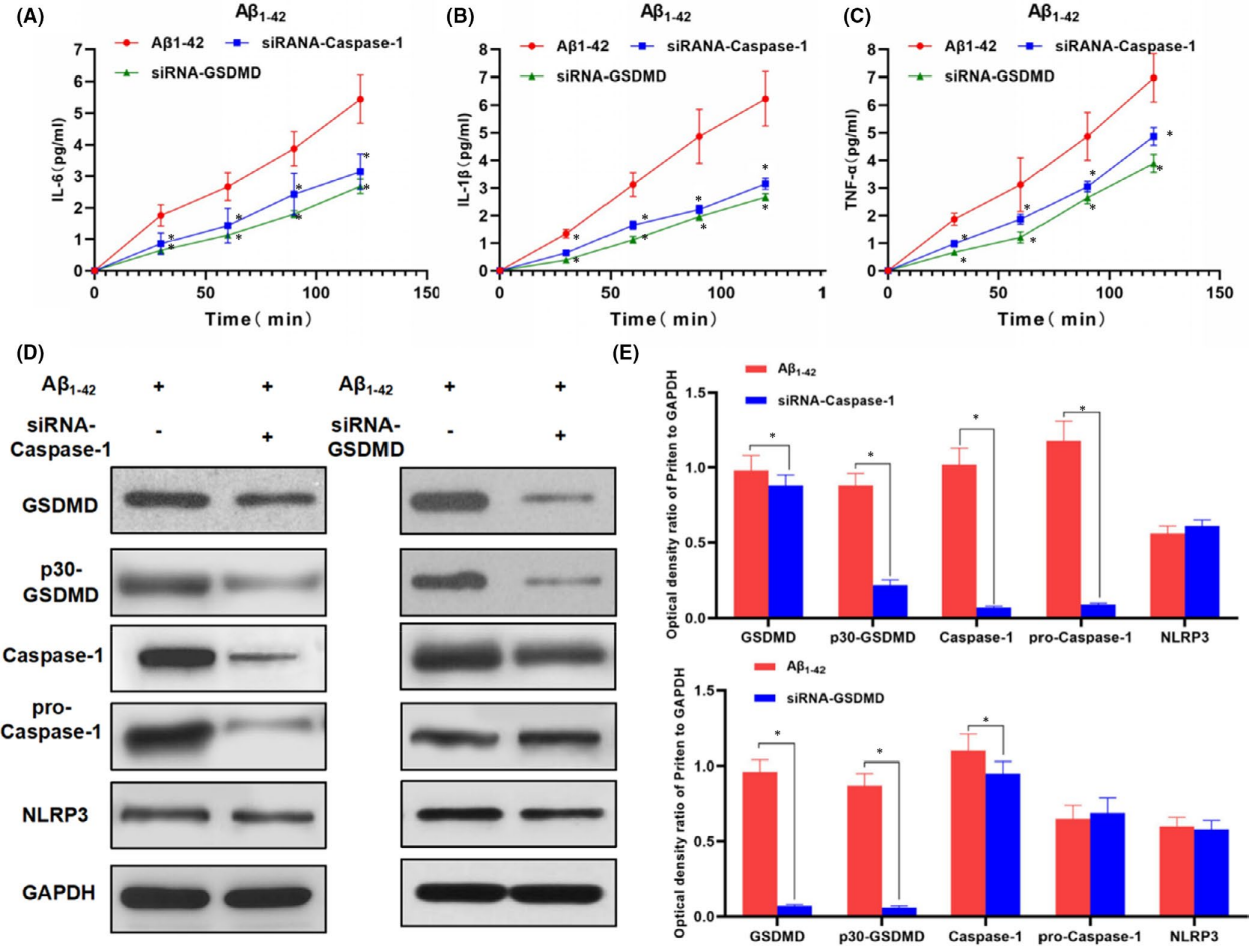
Effects of caspase‐1 or GSDMD silencing on Aβ_1‐42_‐induced inflammatory factor release and expression of pyroptosis‐related protein in MCNs (n = 3). A‐C, The expression levels of inflammatory factors, including IL‐6, IL‐1β and TNF‐α in the culture medium. The expression of inflammatory factors was increased over time in the Aβ_1‐42_ group, indicating increase cell permeability and the release of inflammatory factors. However, the levels of inflammatory factor were significantly down‐regulated in siRNA‐caspase‐1 and siRNA‐GSDMD groups, compared with Aβ1‐42 group, *P < .05. D and E, The expression of pyroptosis‐related proteins. The expression levels of GSDMD and p30‐GSDMD were higher in the Aβ_1‐42_ group. After caspase‐1 inhibition, the level of p30‐GSDMD was down‐regulated, while the expression of GSDMD and NLRP3 was not significantly changed. After suppression of GSDMD, the levels of GSDMD and p30‐GSDMD were lower, but did not affect the expression of upstream cleaved proteins caspase‐1 and NLRP3. Comparison between groups, *P < .05

**FIGURE 6:**
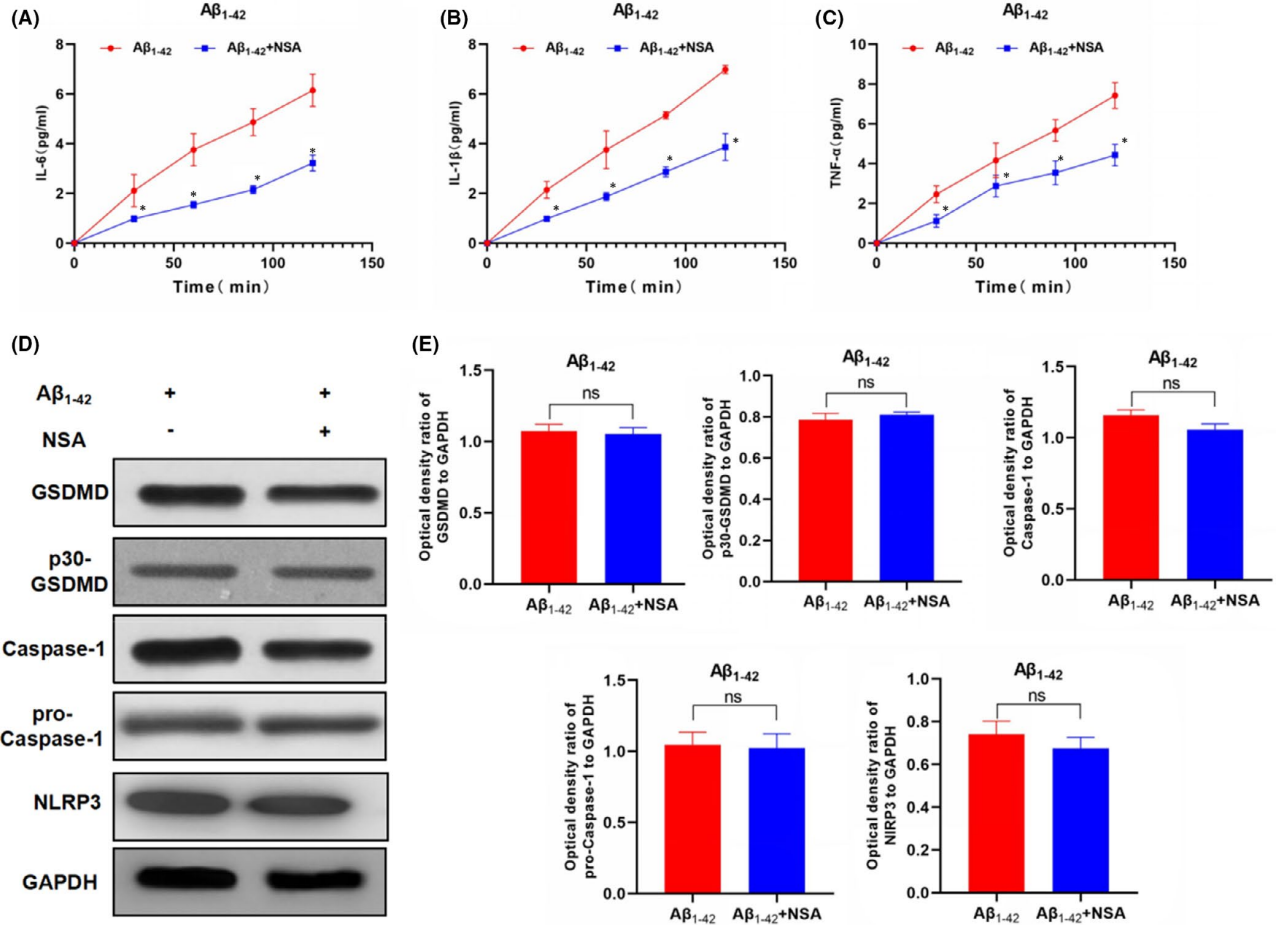
p30‐GSDMD oligomerization inhibitor NSA inhibited Aβ_1‐42_‐induced inflammatory factor release and expression of pyroptotic protein (n = 3). A‐C, The expression levels of inflammatory factors, including IL‐6, IL‐1β and TNF‐α in cell culture medium. The expression of these inflammatory factors was increased over time in the Aβ_1‐42_ group, indicating the increased cell permeability and the release of inflammatory factors. And the levels of inflammatory factors were significantly down‐regulated after NSA intervention, compared with Aβ_1‐42_ group, *P < .05. D and E, The expression level of pyroptosis‐related proteins. The expression levels of GSDMD and p30‐GSDMD (executive protein of pyroptosis) were relatively higher in the Aβ_1‐42_ group; however, the levels of GSDMD and p30‐GSDMD were not significantly changed after NSA intervention. Meanwhile, the expression of caspase‐1 (cleavage protein) or upstream NLRP3 was not significantly changed. Comparison between groups, ^ns^ *P* > .05. These results indicated that NSA did not affect the expression of pyroptosis signalling protein nor did it affect the cleavage, but affected the oligomerization of the effector protein p30‐GSDMD and suppressed the oligomerization of p30‐GSDMD to open the membrane pore
